# Engagement in Care, Awareness, and Interest in Long-Acting Injectable Anti-Retroviral Therapy

**DOI:** 10.1007/s10461-024-04423-x

**Published:** 2024-07-01

**Authors:** Jacob A. Stout, Maxwell Allamong, Frances Hung, Katherine Link, Cliburn Chan, Charles Muiruri, John Sauceda, Mehri S. McKellar

**Affiliations:** 1 Duke University School of Medicine, Durham, NC USA; 2 Duke Initiative on Survey Methodology, Duke University, Durham, NC USA; 3 Department of Biostatistics and Bioinformatics, Duke University School of Medicine, Durham, NC USA; 4 Division of Infectious Diseases, Department of Medicine, Duke University School of Medicine, Durham, NC USA; 5 Department of Population Health Sciences, Duke University, Durham, NC USA; 6 Global Health Institute, Duke University, Durham, NC USA; 7 Center for AIDS Prevention Studies, Division of Prevention Science, Department of Medicine, University of California San Francisco, San Francisco, CA USA; 8 Duke University, P.O. Box 102359, Durham, NC 27710 USA

**Keywords:** HIV, Treatment preferences, Long-acting injectables, Anti-retroviral therapy

## Abstract

Long Acting Injectable (LAI) therapy to treat HIV is an alternative to daily oral medications. The success of early roll-out of LAI to eligible patients requires a better understanding of patients’ awareness and interest in this novel therapy. We administered an electronic survey to patients attending an urban HIV clinic in the US South. Eligible participants were 18 + years old with a most recent HIV-1 viral load < 200 copies/ml, without any evidence of genotypic resistance to LAI components or chronic hepatitis B. Survey recipients were asked about current treatment, engagement in care, and knowledge of LAI. Between January-April 2023, 480 patients were screened; 319 were eligible, and 155 (49%) completed the survey. The majority (119, 77%) were aware of, and 87 (56%) were interested in LAI. In regression analysis, only age was associated with interest in LAI (OR 0.95, 95% CI 0.92,0.99). Among proposed benefits of injectables, ease of travel without pills, lack of daily pill-taking, and fewer medication interactions were most appealing. Among proposed concerns with injectables, higher cost and insurance coverage of the new medicine were most worrisome. A large majority of people with HIV (PWH) are aware of the newest treatment available, and just over half of our sample expressed interest in LAI. Older age was associated with lower interest in LAI. LAI is appealing for its convenience, privacy, and avoidance of drug interactions, while the increased costs associated with LAI need to be addressed.

**Supplementary Information:**

The online version contains supplementary material available at 10.1007/s10461-024-04423-x.

## Introduction

A new approach to chronic treatment of HIV has come to market in the form of long acting injectable (LAI) medication. In 2024, there is one FDA-approved option for a complete LAI regimen consisting of the integrase strand transfer inhibitor (INSTI) cabotegravir (CAB) and the non-nucleoside reverse transcriptase inhibitor (NNRTI) rilpivirine (RPV). Notably, in January 2022 the FDA expanded the CAB + RPV label for dosing every 8 weeks rather than 4 weeks, reducing the burden of LAI-related clinic visits and thereby making CAB + RPV a more convenient option for those patients interested in switching off oral anti-retroviral therapy (ART) [1–3].

There has been interest in LAI alternatives to oral medications among people with HIV (PWH) and HIV care providers for both prevention and treatment of HIV for years, with an increase in published data on the topic as clinical trials progressed [4–6]. Previous studies document both excitement about the prospect of a new long-acting treatment option as well as hesitation to adopt the new treatment due to concerns about its novelty, its efficacy, or the logistical barriers to receiving regular injections [7–11]. Proposed benefits of LAI have included both convenience and privacy for people who are well controlled on oral medications, in addition to the hypothesized benefit of improved medication adherence for those who struggle to consistently take daily pills [9, 10, 12–14]. In the LATTE, ATLAS, FLAIR, and ATLAS 2-M trials, patient reported outcomes showed an overwhelming preference for LAI over participants’ previously utilized oral medications [15–18]. However, there are concerns about generalizability of trial results to the greater population of PWH as the analyzed data came from a subset of PWH who were willing to enroll in a LAI clinical trial. While implementation studies are ongoing, including the LATITUDE trial focusing particularly on individuals with a history of medication non-adherence (NCT03635788), data are lacking about the real-world reception of LAI among PWH.

For our study, we surveyed participants’ preferences regarding LAI CAB + RPV. We hypothesized that a patient’s engagement in HIV care would be associated with greater awareness and interest in LAI-ART. Additionally, this study aims to provide initial data regarding respondents’ awareness, interest, and concerns about LAI outside of a clinical trial setting and among a sample of PWH that is more representative of our clinic population. This allows better understanding of who will benefit most from LAI to guide best utilization of the new therapy. These data are especially important given our clinic location in the US South, an area disproportionately impacted by HIV incidence, morbidity, and mortality [19].

## Methods

This was a cross-sectional survey study that recruited participants using a convenience sampling approach at Duke Infectious Diseases Clinic in Durham, North Carolina. Participants consisted of patients presenting for routine HIV care from January-April 2023. Inclusion criteria included being an adult (18 + years old) living with HIV with a suppressed HIV-1 viral load (< 200 copies/mL) on oral ART with no evidence of NNRTI or INSTI resistance. Exclusion criteria included a detectable HIV-1 viral load (> 200 copies/mL), any evidence of resistance to NNRTI or INSTI medications, chronic hepatitis B, non-English speaking, or poor candidacy (described below). Research personnel were present in clinic on the three days each week with the highest volume of HIV care providers and would screen the schedules of all HIV providers for eligible participants. When an eligible individual checked in for their appointment, their provider was made aware of the individual’s eligibility for survey participation. The provider then allowed us to approach the patient for participation before or after the clinical visit, asked the patient directly if they would participate, or requested that the survey not be offered to that individual (i.e. poor candidacy). The definition of poor candidacy was left to the primary HIV provider’s discretion, but participants who met that criterion were excluded by their provider due to being unlikely to agree to participation (e.g. declined research participation previously) and/or having elevated psychosocial stressors identified by their HIV provider such that requesting their participation in a study might be inappropriate. Surveys were administered electronically using Qualtrics^®^, and participation required approximately 10 min. Respondents had the option to complete the survey in clinic during their appointment on iPads or desktop computers. Alternatively, they could use a QR code or link sent to their email or electronic medical record portal to complete the survey on their own devices. When needed or requested, research personnel administered the survey verbally to those with barriers to completing it independently. Participants who completed the survey could elect to participate in a raffle for one of five $50 gift cards. The study was approved by Duke University Health System’s Institutional Review Board (Pro00111870).

Clinical and demographic information were collected. The survey was developed in collaboration with the Duke Initiative for Survey Methodology and consisted of three sections aimed at understanding participants’ experience with their oral medications, their engagement in care, and their knowledge of LAI. The first section addressed respondents’ understanding of their current medication regimen and probed their side-effects. It also gave the opportunity to name anything a participant may want to change about their HIV treatment. Section one concluded with a question about one’s likelihood of changing medicines at the next visit. This question came before any mention of LAI had been introduced to the participant. The second section included the Brief HIV Index– a 3-item tool derived from the Index of Engagement in HIV Care that yields a numerical score correlating with one’s perceived level of engagement in, and connection to, their HIV care [20–22]. The original scale has 10 items, but the short form utilized in this study has also been validated to predict future retention in care outcomes [23]. The Brief HIV Index is comprised of the following three questions:

1. How open do you feel you can be with your HIV provider?
2. How often do you leave your HIV care appointment feeling like you got really good care?
3. How well do you follow through on your HIV care when things in your life get tough?

Responses to each question are scored from 1 to 5, with 5 indicating the highest level of engagement. The participant’s engagement score is the average of their responses to the three questions.

The third section of the survey assessed participants’ awareness, interest, and concerns with LAI. We provided the following educational statement immediately after asking respondents about their awareness of LAI, but before asking about their interest or concerns:

“Injectable medicines for HIV, also called Long-Acting Injectables or LAI, are a new treatment option for HIV that is an alternative to daily pills for some patients. If your HIV care provider determines that you qualify, you can come to the HIV clinic to receive two injections– one injection in each gluteus muscle (each side of the butt)-- every 1 to 2 months.”

After asking about awareness of LAI and providing the above educational statement, respondents were asked to rate their interest in receiving their medicine by injection if they were to be eligible. Next, they rated their individual level of appeal or concern associated with specific benefits (e.g. travelling without pills) or drawbacks (e.g. insurance coverage of new medication) of injectable medicines. The final question reminded participants of their previously expressed likelihood of changing medicines at the next visit (end of section one) and asked them to rate their likelihood of changing to injectables after having considered the features of LAI. A side-by-side bar plot was then used to visualize change in a participant’s likelihood of changing HIV medication before and after learning about and considering LAI.

Descriptive statistics– including frequency and percent for categorical data and median, Q1, and Q3 for continuous data– were used to summarize the data. To assess the association between selected covariates and participant awareness of LAI, a multivariable logistic regression (N = 145) was fit using race, ordinalized education, and engagement score. A multivariable logistic regression for interest in LAI (N = 116) was fit using race, age, side effects, and awareness of LAI. Participants who reported neutral interest—“neither interested nor disinterested” in LAI—were excluded. In both regression models, BCa (bias-corrected and accelerated) confidence intervals were calculated for estimated regression coefficients. Two survey questions (year that patient started current medication and year that patient established with current HIV Provider) are not reported in the manuscript due to inaccuracy in the answers. Participants with incomplete responses were excluded from the models, and the data used in all analyses were unweighted.

Respondents were also grouped by expressed interest, neutrality, or lack of interest in LAI for a descriptive comparison of their clinical and demographic characteristics. To learn which specific factors of LAI are of benefit or concern to respondents (i.e. more privacy, ease of travel, insurance coverage, fear of needles, etc.), the responses for each factor were coded from 0 to 3 (0 = not at all appealing/concerning to 3 = extremely appealing/concerning). Mean self-ascribed importance of potential benefits and concerns was visualized in a dumbbell plot, colored by whether participants were interested in LAI or not. Our data set, while not publicly available, can be made available upon request.

## Results

During the study period, 480 individuals with HIV were screened; 319 were found to be eligible, and 155 (49%) completed the survey (Supplemental Fig. 1). Sixteen individuals (3.3% of screened) were excluded due to not speaking English. 25% of participants were female; 44% Black, 7% Latinx; mean age was 48, with ages ranging from 21 to 77.

### Current Treatment

85% reported currently taking one pill daily for HIV, and 75% of participants denied experiencing side effects from their HIV medicine. Details of reported side effects are in Table 1, as well as a summary of clinical and demographic characteristics. Twenty-four participants used the free text option to report something that they would like to change about their current treatment. Of those, 12 desired non-daily dosing of medication, five specifically stated they wanted to be on injectables, and two desired less weight gain. The remaining changes desired by one participant each were better insurance coverage, a single injection regimen, less diarrhea, less nausea, and less liver toxicity. Overall, participants were considered highly engaged in care using the Brief HIV Index (Mean 4.6/5, SD: 0.5).

**Table 1:** Clinical and demographic characteristics

Characteristic	*N* = 155
**Age**	
Mean (SD)	48.4 (14.1)
Median (Q1, Q3)	49 (37, 60)
Range	(21, 77)
**Gender**	
Male	113 (72.9%)
Female	38 (24.5%)
Transgender female	3 (1.9%)
Prefer not to answer	1 (0.6%)
**Hispanic or Latino**	
Yes	10 (6.5%)
No	138 (89.0%)
Prefer not to answer	4 (2.6%)
Missing	3 (1.9%)
**Self Described Race**	
White	66 (42.6%)
Black or African American	68 (43.9%)
American Indian or Alaska Native	2 (1.3%)
Asian or Asian American	2 (1.3%)
Native Hawaiian or other Pacific Islander	1 (0.6%)
Some other race or origin (please specify)	7 (4.5%)
Prefer not to answer	6 (3.9%)
Missing	3 (1.9%)
**Level of Education**	
Completed college degree	71 (45.8%)
Some college, but not degree	45 (29.0%)
High school degree or equivalent	37 (23.9%)
Less than high school	2 (1.3%)
**Relationship Status**	
Single	86 (55.5%)
Partnered	65 (41.9%)
Prefer not to answer	4 (2.6%)
**Sexual Activity (in last 6 months)**	
Sexually active, no new partners	59 (38.1%)
Sexually active, one or more new partners	37 (23.9%)
Not sexually active	52 (33.5%)
Prefer not to answer	7 (4.5%)
**Years Since Diagnosis**	
Mean (SD)	15.3 (10)
Median (Q1, Q3)	13 (8, 22)
Range	(1, 40)
**Current Treatment**	
One pill per day	131 (84.5%)
More than one pill per day	24 (15.5%)
**Any Side Effects**	
Yes, I experience side effects	30 (19.4%)
No, I don’t experience side effects	116 (74.8%)
I don’t know if I experience side effects	9 (5.8%)
**Side Effect: Nausea/vomiting** ^a^	
Extremely bothersome	1 (0.6%)
Moderately bothersome	3 (1.9%)
Slightly bothersome	9 (5.8%)
I do not experience this side effect	26 (16.8%)
**Side Effect: Diarrhea**	
Extremely bothersome	5 (3.2%)
Moderately bothersome	5 (3.2%)
Slightly bothersome	7 (4.5%)
I do not experience this side effect	22 (14.2%)
**Side Effect: Abdominal pain**	
Extremely bothersome	2 (1.3%)
Moderately bothersome	6 (3.9%)
Slightly bothersome	9 (5.8%)
I do not experience this side effect	22 (14.2%)
**Side Effect: Headaches**	
Extremely bothersome	1 (0.6%)
Moderately bothersome	6 (3.9%)
Slightly bothersome	6 (3.9%)
I do not experience this side effect	26 (16.8%)
**Side Effect: Weight gain**	
Extremely bothersome	8 (5.2%)
Moderately bothersome	6 (3.9%)
Slightly bothersome	4 (2.6%)
I do not experience this side effect	21 (13.5%)
**Side Effect: Abnormal dreams/nightmare**	
Extremely bothersome	3 (1.9%)
Moderately bothersome	1 (0.6%)
Slightly bothersome	8 (5.2%)
I do not experience this side effect	27 (17.4%)
**Side Effect: Insomnia**	
Extremely bothersome	3 (1.9%)
Moderately bothersome	5 (3.2%)
Slightly bothersome	14 (9.0%)
I do not experience this side effect	17 (11.0%)
**Side Effect: Depression or feeling sad**	
Extremely bothersome	1 (0.6%)
Moderately bothersome	3 (1.9%)
Slightly bothersome	6 (3.9%)
I do not experience this side effect	29 (18.7%)
**Side Effect: Weight loss**	
Extremely bothersome	1 (0.6%)
Moderately bothersome	3 (1.9%)
Slightly bothersome	1 (0.6%)
I do not experience this side effect	34 (21.9%)
**Side Effect: other (please specify)** ^b^	
Extremely bothersome	2 (1.3%)
Moderately bothersome	2 (1.3%)
Slightly bothersome	4 (2.6%)
I do not experience this side effect	31 (20.0%)
**Told a medicine doesn’t work for you**	
Yes, I’ve been told a med doesn’t work	25 (16.1%)
No, I haven’t been told a med doesn’t work	124 (80.0%)
I don’t know	5 (3.2%)
**Aware of Injectables**	119 (76.8%)
**Interested in Injectables**	
Very interested	35 (22.6%)
Slightly interested	52 (33.5%)
Neither interested nor disinterested	22 (14.2%)
Slightly disinterested	17 (11.0%)
Very disinterested	28 (18.1%)
**Engagement Score**	
Mean (SD)	4.6 (0.5)
Range	(1.7, 5)

a. Specific side effect questions were only answered by the 39 individuals who answered that they either had side effects or did not know if they had side effects

b. Other side effects reported included hunger (1), sleepiness (1), constipation (1), neuropathy (1), dizziness (1), and “no weight loss” (1). The remaining two respondents who reported being bothered by “other” side effects did not specify the side effect in the provided free-text box

### Awareness of LAI

**Fig. 1:**
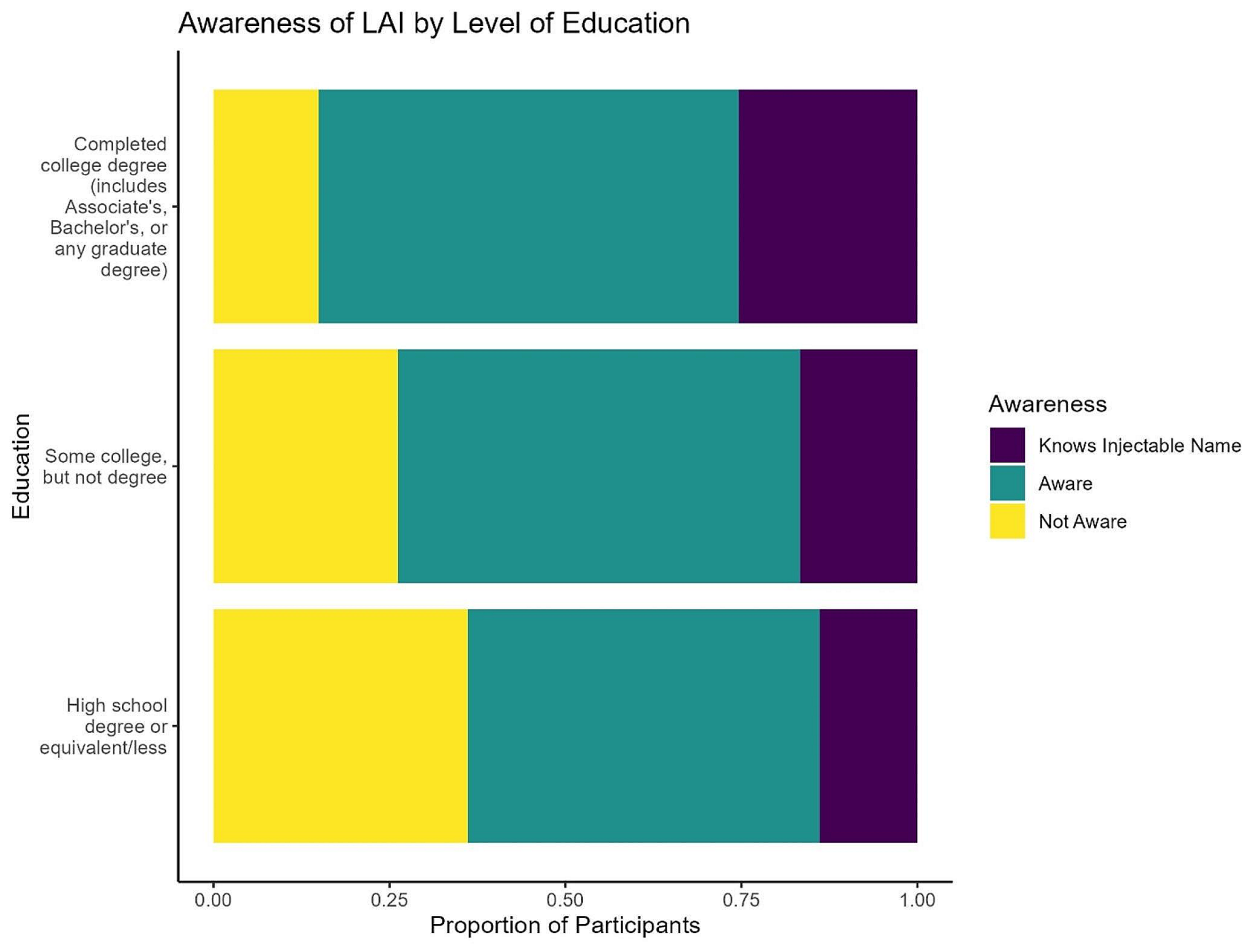
This figure depicts the relationship between level of education and participants’ awareness of LAI as a treatment option for HIV. Response options were the following: not aware of injectables, aware of injectables but unable to accurately name medication, and aware of injectables and able to accurately name medication

The majority (119, 77%) of respondents were aware of injectable treatments. The data suggest that education may be positively correlated with awareness, but results were not statistically significant (Fig. 1; stacked bar plot). None of the covariates in the regression model were associated with awareness of LAI (Table 2).

**Table 2:** Results of logistic regression of awareness of LAI

Term	Odds Ratio (95% CI)
Race	
White	Reference
Black	1.29 (0.52,3.2)
Underrepresented Minorities^a^	0.24 (0.01,4.48)
Education (Ordinal)	1.57 (0.91,2.72)
Engagement Score	1.55 (0.7,3.43)

a. Aggregate of all racial groups that are not black or white, excluding those that did not provide race data. See Table 1 for included groups

### Interest in LAI

**Fig. 2:**
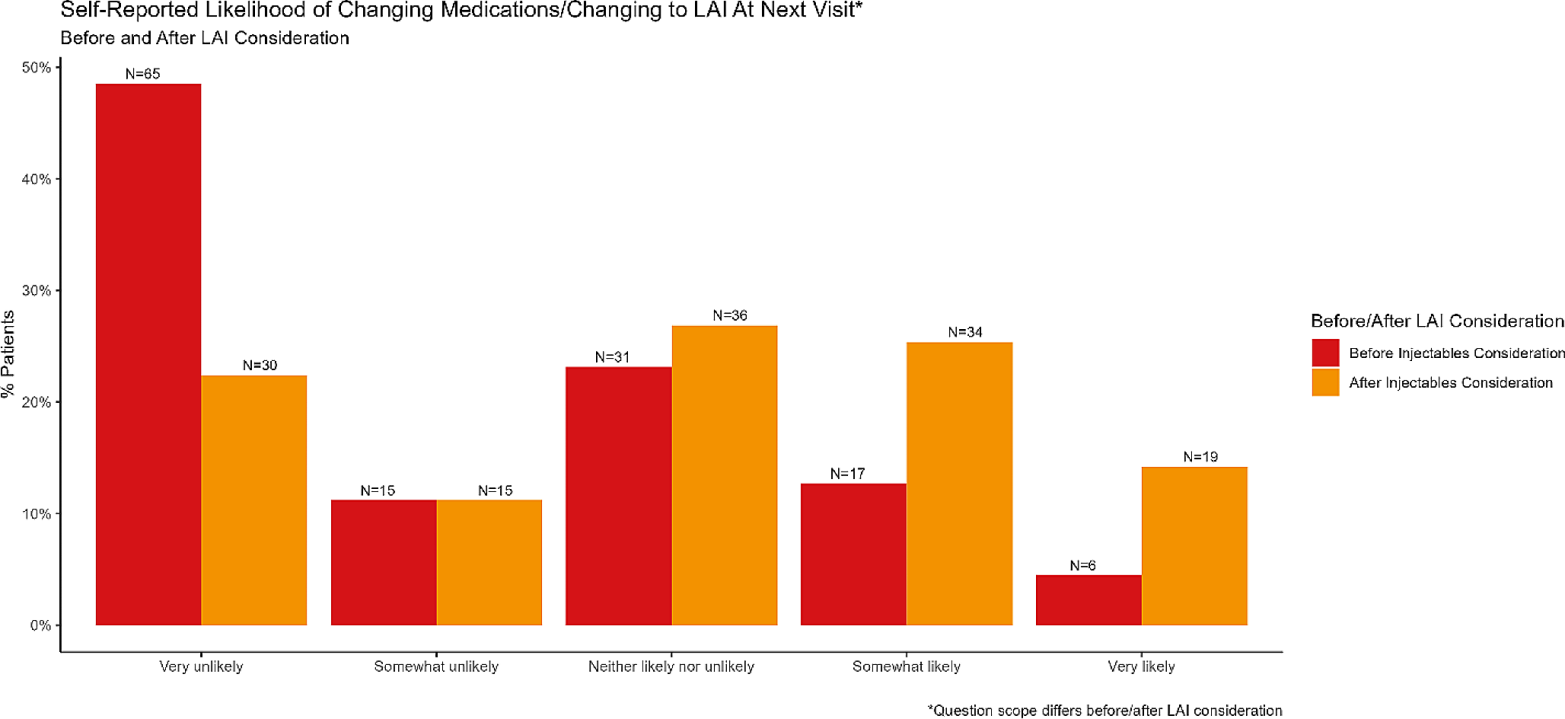
The response rates are summarized for the questions asking participants to rate their likelihood of changing medication at their next visit. Notably, the first question asked participants about their likelihood of changing medicines generally, while the second question asked specifically about changing to LAI at the next visit

Over half (87, 56%) of participants demonstrated interest in injectable treatments by responding that they were either “Very” or “Slightly” interested. Age was the only factor associated with interest in LAI in the regression model, with higher age being associated with a lower likelihood of interest in LAI (OR 0.95, 95% CI 0.92,0.99; Table 3). In a descriptive comparison between individuals interested and not interested in LAI, interested respondents were younger and were, on average, more recently diagnosed with HIV (Table 4). Engagement scores were lower in the group that was interested in LAI as compared to those uninterested in LAI (Mean 4.5, SD 0.5 vs Mean 4.6, SD 0.7, respectively). As such, higher engagement in care was felt not to be associated with higher interest in LAI and was therefore excluded from regression analysis. Finally, interest in changing medications increased after consideration of LAI (Fig. 2; side-by-side bar plot).

**Table 3:** Results of logistic regression of interest in LAI

Term	Odds Ratio (95% CI)
Race	
White	Reference
Black	1.95 (0.75,5.07)
Underrepresented Minority^a^	0.66 (0,167.98)*
Age	0.95 (0.92,0.99)
Side Effects	1.19 (0.25,5.66)
Awareness of LAI	0.75 (0.24,2.33)

a. Aggregate of all racial groups that are not black or white, excluding those that did not provide race data. See Table 1 for included groups

* Effect should not be interpreted due to limited sample size

**Table 4:** Characteristics of respondents by level of interest in LAI

Characteristic	Interested *N* = 87	Neutral *N* = 22	Uninterested *N* = 45
**Age**			
Mean (SD)	45.4 (13.7)	46.3 (14)	55.3 (12.6)
Median (Q1, Q3)	44 (34, 57)	47.5 (34.2, 55)	58 (49, 65)
Range	(21, 77)	(21, 69)	(22, 74)
**Gender**			
Male	64 (73.6%)	19 (86.4%)	29 (64.4%)
Female	20 (23.0%)	3 (13.6%)	15 (33.3%)
Transgender female	2 (2.3%)	0 (0.0%)	1 (2.2%)
Prefer not to answer	1 (1.1%)	0 (0.0%)	0 (0.0%)
**Self Described Race**			
White	31 (35.6%)	13 (59.1%)	22 (48.9%)
Black or African American	45 (51.7%)	7 (31.8%)	15 (33.3%)
American Indian or Alaska Native	1 (1.1%)	0 (0.0%)	1 (2.2%)
Asian or Asian American	1 (1.1%)	0 (0.0%)	1 (2.2%)
Native Hawaiian or other Pacific Islander	1 (1.1%)	0 (0.0%)	0 (0.0%)
Some other race or origin (please specify)	3 (3.4%)	1 (4.5%)	3 (6.7%)
Prefer not to answer	4 (4.6%)	0 (0.0%)	2 (4.4%)
Missing	1 (1.1%)	1 (4.5%)	1 (2.2%)
**Level of Education**			
Completed college degree	41 (47.1%)	12 (54.5%)	18 (40.0%)
High school degree or equivalent	19 (21.8%)	6 (27.3%)	11 (24.4%)
Less than high school	0 (0.0%)	0 (0.0%)	2 (4.4%)
Some college, but not degree	27 (31.0%)	4 (18.2%)	14 (31.1%)
**Relationship status**			
Single	53 (60.9%)	9 (40.9%)	23 (51.1%)
Partnered	31 (35.6%)	13 (59.1%)	21 (46.7%)
Prefer not to answer	3 (3.4%)	0 (0.0%)	1 (2.2%)
**Sexual Activity (6 months)**			
Sexually active, no new partners	32 (36.8%)	9 (40.9%)	18 (40.0%)
Sexually active, one or more new partners	19 (21.8%)	10 (45.5%)	8 (17.8%)
Not sexually active	29 (33.3%)	3 (13.6%)	19 (42.2%)
Prefer not to answer	7 (8.0%)	0 (0.0%)	0 (0.0%)
**Years Since HIV Diagnosis**			
Mean (SD)	13.3 (9.6)	14.6 (8.8)	19.6 (10.2)
Median (Q1, Q3)	11 (7, 18.8)	16.5 (7.2, 18.8)	21 (10, 27)
Range	(1, 40)	(1, 34)	(2, 38)
**Side Effects (Any)**			
Yes, I experience side effects	15 (17.2%)	6 (27.3%)	9 (20.0%)
No, I don’t experience side effects	66 (75.9%)	15 (68.2%)	34 (75.6%)
I don’t know if I experience side effects	6 (6.9%)	1 (4.5%)	2 (4.4%)

### Benefits/Concerns

**Fig. 3:**
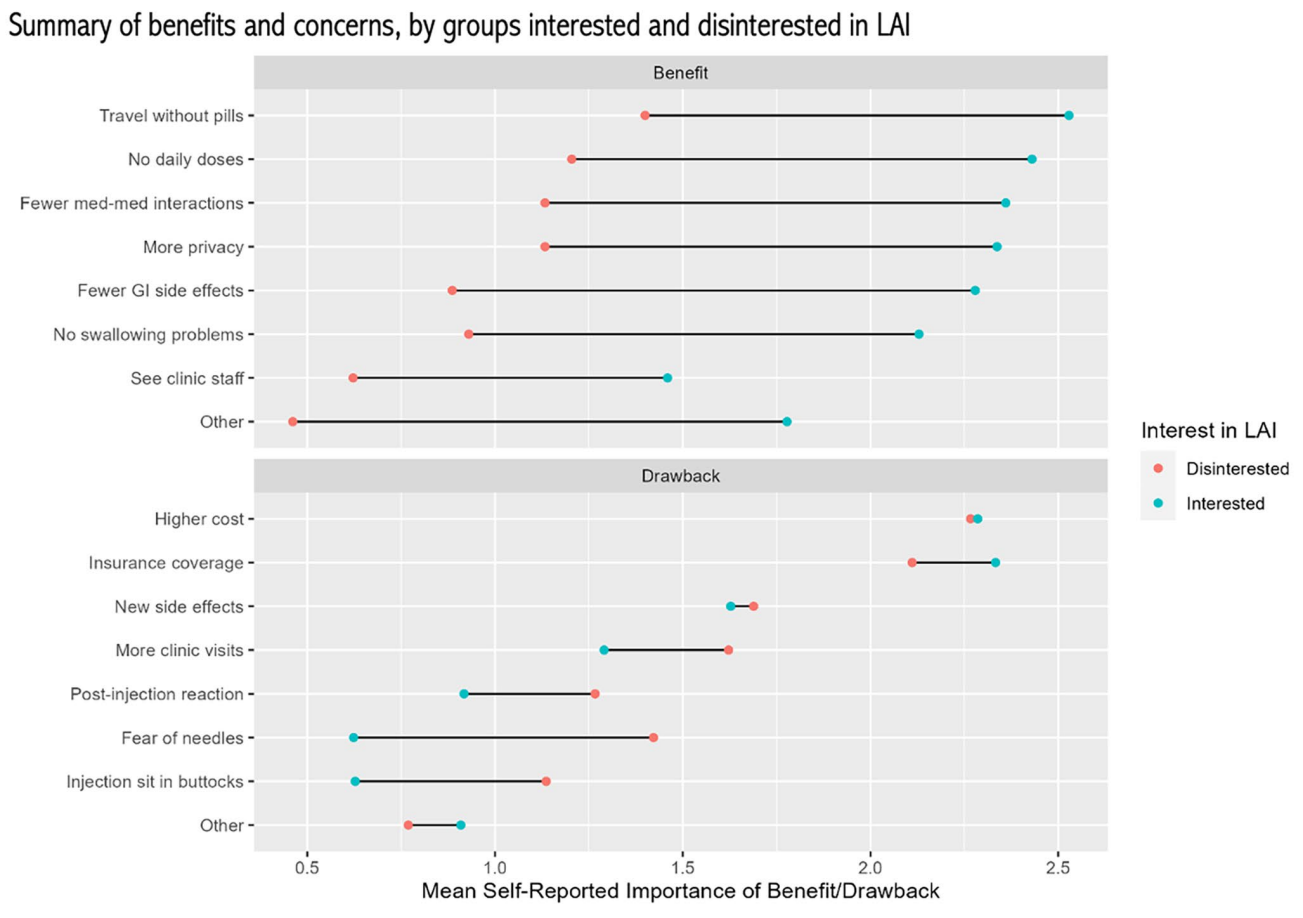
Participants were asked to rate the appeal or concern associated with each of the listed factors related to LAI. Responses options were 0 = Not at all appealing/worrisome, 1 = Slightly appealing/worrisome, 2 = Moderately appealing/worrisome, 3 = Extremely appealing/worrisome. The average rating for each factor was calculated among individuals interested in LAI and those disinterested for comparison between groups

The LAI factors that participants found to be most appealing included ease of travel without pills, fewer medication interactions, and lack of daily doses. The dominant concerns with LAI were the insurance coverage and out-of-pocket cost of the new drug, while the possibility of new side effects was also of concern. Figure 3 summarizes participants’ attitudes toward the benefits and concerns that were included in the survey, grouped by those interested (“Slightly” or “Very” interested) and not interested (“Slightly” or “Very” disinterested) in LAI. While interested participants consistently rated the benefits of LAI as more appealing than did their disinterested counterparts, all patients demonstrated high concern for the cost associated with changing therapy.

## Discussion

This survey-based study provides preliminary data on the perspectives of PWH in the US South regarding LAI. Overall, more than ¾ of survey participants were aware of LAI, and more than half were interested in LAI. While clinical trials have shown > 90% of patients on LAI prefer injectables over previously used pills, our findings are similar to other real-world studies among PWH showing 57–66% are interested in switching from oral medicines to LAI [13, 17, 18, 24, 25], or to a non-daily treatment regimen [26]. One recent study showed only 31% of patients to be accepting of LAI with monthly dosing, but with the larger interval between doses at the time of our study, a higher level of interest is expected [27]. Engagement in care, as measured by the Brief HIV Index, was not associated with individuals’ awareness or interest in LAI [23]. It is worth noting that our recruitment strategy required attendance at clinic appointments, likely selecting for more engaged participants and introducing a ceiling effect on engagement scores in our sample. Future implementation studies should seek to include participants with lower engagement scores to detect the predictive power of patient engagement more effectively.

While it is encouraging that most respondents were aware of LAI, our findings raise concern that individuals with lower educational attainment may be disadvantaged by lack of awareness of new treatments. Other data have also shown a lower education level is associated with preference to stay on daily oral therapy rather than transition to any long-acting option [28]. In light of these findings, low education appears to be a contributor to low uptake of LAI. The exact mechanism of that effect is not fully understood and is likely multifactorial. Even as long-acting therapies advance, there will continue to be an important market for daily oral medications. Additionally, patient education on the availability of new therapies will be important for optimal uptake.

Those who were interested in LAI were more often younger and were, on average, more recently diagnosed. That said, the average time since diagnosis in the interested group was 13 years, suggesting that early treatment decisions in the first years after diagnosis are not the only opportunity for patients to be interested in LAI. Additionally, in the regression analysis, older age was associated with lower interest in LAI. The findings regarding age and time of diagnosis are consistent with what was found by Akinwunmi et al. (2021) in a European sample and Simoni et al. (2019) in a US Northwest sample, while Barthold et al. (2023) also found a similar relationship with age in a sample including the US South. This suggests that those trends are not limited to a geographical setting. That said, some proposed benefits of LAI are advantageous for an aging person, particularly those with a large pill burden. While increasing age is associated with lower interest in LAI overall, our data do not rule out the possibility of LAI appealing to older adults with specific barriers (i.e. dysphagia, polypharmacy, interactions) to oral medication adherence and efficacy [14].

The benefits most appreciated and concerns most worrisome according to the participants in our study were similar to those seen in previous literature [7, 10, 13, 14, 25, 27, 29]. Namely, those interested in LAI appreciate the convenience of injections (easy travel, no missed doses), the privacy afforded by LAI, and the avoidance of gastrointestinal side effects or medication interactions. However, interested and disinterested people alike frequently (~ 75% overall) rated themselves “moderately” or “extremely” concerned about cost or insurance coverage of LAI. Previous literature on LAI for HIV treatment has largely been conducted in a cost-neutral environment, and so it is important to note how cost impacts treatment decisions as cost has been identified as a barrier to transitioning to LAI [30]. A recent study in Australia also found out-of-pocket cost to be a key determinant of ART preference, while a pilot study in the US South noted delays in treatment initiation due to insurance denials [31, 32]. This is particularly concerning in the US healthcare market with the complex mix of private and public payors. A unique feature of LAI access in our healthcare system is the division of individuals by insurance status- in our clinic, uninsured PWH have easier and more affordable access to new HIV medicines through the AIDS Drug Assistance Plan. Meanwhile, those with private insurance often have a high financial barrier to accessing these medicines while insurance companies and health systems learn how to bill and reimburse for a new drug and treatment modality. It is common for the injections to only be covered under medical benefits, introducing higher cost sharing requirements than would be expected if the medication were covered by pharmacy benefits. We did not collect data on insurance status in this study, but an investigation of preference variability and accessibility of the medication by insurance status is an area for future research as differences in insurance coverage may contribute to disparities in uptake.

The strength of this study is its ability to give a real-world assessment of the reception of LAI among PWH. That is because all participants were eligible for LAI as a prerequisite for taking the survey, making it a realistic option for them to consider. We also did not assume cost neutrality, allowing us to highlight the importance of financial concerns among survey respondents. A limitation in this study is its reliance on participant reported data, introducing the possibility of reporting bias. While Hispanic and Spanish speaking individuals comprise an important (and growing) group of PWH in the US, we did not develop a Spanish language version of the survey. Only 6.5% of respondents were Hispanic or Latino, and only 3.3% of screened patients were excluded based on language. As such, our clinic’s demographics do not allow our findings to be generalizable to the Hispanic or Spanish-speaking population, and further study should investigate the treatment preferences of those patients. Additionally, our inclusion criteria’s focus on patients eligible for LAI was restrictive, omitting a portion of patients who could soon become eligible for LAI despite being technically ineligible on the day of their visit to clinic. Even with a strong sample size, some of the variables in the regression models had small sub-groups. Finally, we only surveyed individuals who presented for care and were compliant on oral medications, making us unable to understand the preferences of less engaged persons who may benefit most from a treatment alternative that putatively improves adherence.

## Conclusions

A large majority of PWH are aware of the newest treatment modality available to them, and just over half of the participants in our sample expressed interest in LAI. Age was statistically associated with interest in LAI, with older patients being less interested. LAI is appealing for its convenience, privacy, and avoidance of drug interactions, while the increased costs associated with LAI need to be addressed.

## Electronic Supplementary Material

Below is the link to the electronic supplementary material.

Supplementary Material 1

Supplementary Material 2
