# Supplementary material for: Engagement in Care, Awareness, and Interest in Long-Acting Injectable Anti-Retroviral Therapy
Source: AIDS Behav. 2024 Jul 1;28(10):3315–25. doi: 10.1007/s10461-024-04423-x (PMC11427500; doi:10.1007/s10461-024-04423-x)
Supplement: Supplementary file 1 — Supplementary Material 1 [file 10461_2024_4423_MOESM1_ESM.pdf]

Consent

Thank you for taking part in this survey. We want to learn how patients living with HIV think about their care and the medicine they take for HIV.

This survey will take about 10 minutes to complete, although the length can be different depending on your own speed. Your responses to this survey are confidential and you will remain anonymous. You can join a raffle for one of five $50 Amazon gift cards by providing you contact information at the end of the survey. Contact information for the raffle will be kept separate from your answers to the survey questions.

Your participation is voluntary. You may choose to stop participating at any time. If you wish to stop, simply close your browser window. There is minimal risk to you in completing this survey. If you have any questions about the survey, you may contact Jacob Stout (jacob.stout@duke.edu) or Dr. Mehri McKellar (mehri.mckellar@duke.edu) in Clinic 1K.

By selecting 'I Agree' below you are agreeing that you have read the above and are willing to participate in the survey.

I Agree

I Do Not Agree

Current Treatment

What is your current treatment for HIV, if any at all?

One pill per day

More than one pill per day

Injectable medicines

None- I do not take medicine for HIV

Other

Do you know the result of your most recent laboratory tested HIV viral load?

Yes, I know the result

No, I don't know the result

In what year were you diagnosed with HIV? If you are unsure of the exact year, please take a guess at the approximate year.

In what year did you start your current HIV medicine? If you are unsure of the exact year, please take a guess at the approximate year.

In what year did you start seeing your current HIV care provider? When referring to your "HIV care provider" we are asking about the doctor, physician assistant (PA), or nurse practitioner (NP) that you feel is your primary provider of HIV-related care.If you are unsure of the exact year, please take a guess at the approximate year.

Do you remember your HIV care provider telling you that a medicine does not work for you (including being told you have resistance to an HIV medicine)?

Yes, I have been told a medicine doesn't work for me

No, I have not been told that a medicine doesn't work for me

I don't know if I have been told that a medicine doesn't work for me

When thinking about your current HIV medicine(s), how important, if at all, are each of the following factors when you and/or your HIV care provider are deciding which medicine is right for you?

	Not at all important	Slightly important	Moderately important	Very important	Extremely important	Unsure
Insurance coverage	○	○	○	○	○	○
Out-of-pocket cost	○	○	○	○	○	○
Effects of my HIV medicine on the other medicines I take	○	○	○	○	○	○

	Not at all important	Slightly important	Moderately important	Very important	Extremely important	Unsure
Number of pills I take in a day	○	○	○	○	○	○
Size of the pill(s)	○	○	○	○	○	○
Side effects of the medicine	○	○	○	○	○	○

Do you feel that you experience side-effects from your current HIV medicine?

Yes, I experience side effects

No, I don't experience side effects

I don't know if I experience side effects

Listed below are several possible side effects of HIV medicines. Please rate each side effect according to how much it bothers you, if at all. If you do not feel that you experience a side-effect that is listed, or if that side-effect is caused by a different medicine you take besides HIV medicine, please select "I do not experience this side effect from my HIV medicine."

	Slightly bothersome	Moderately bothersome	Extremely bothersome	I do not experience this side effect from my HIV medicine
Abnormal dreams and/or nightmares	○	○	○	○
Weight gain	○	○	○	○
Diarrhea	○	○	○	○
Headaches	○	○	○	○

	Slightly bothersome	Moderately bothersome	Extremely bothersome	I do not experience this side effect from my HIV medicine
Depression and/or feeling sad	○	○	○	○
Abdominal pain	○	○	○	○
Nausea/vomiting	○	○	○	○
Insomnia and/or inability to sleep	○	○	○	○
Weight loss	○	○	○	○
Side effect not listed here (please specify):	○	○	○	○

Is there anything you wish to change about your HIV medicines?

Yes

No, there is nothing I wish to change

What would you change?

How likely or unlikely are you to make a change to the medicine(s) you take for HIV at your

next visit?

Very unlikely

Somewhat unlikely

Neither likely nor unlikely

Somewhat likely

Very likely

I Don't Know

Abbreviated Index of Engagement in HIV care

Next, we have just three questions about your HIV care team and your HIV care in general. Please remember that when referring to your "HIV provider" we are asking about the doctor, physician assistant (PA), or nurse practitioner (NP) that you feel is your primary provider of HIV-related care. As stated previously, all your responses will remain confidential. The individuals results of this survey will not be shared with your care team.

How open do you feel you can be with your HIV care provider?

Not at all open

Slightly open

Moderately open

Very open

Extremely open

How often do you leave your HIV care appointment feeling like you got really good care?

Never

Sometimes

About half the time

Most of the time

Always

How well do you follow through on your HIV care when things in your life get tough?

Not at all well

Slightly well

Moderately well

Very well

Extremely well

Long-Acting Injectable Medicine Questions

Now we have a set of questions about your potential HIV treatment options that may or may not be different from the treatment you currently use.

Are you aware that injectable medicines exist for HIV?

Yes

No

If you can, please provide the name (generic or brand name) of the medicine you are aware of. If you do not know the name of the medicine, please type N/A in the text box.

<ins>LAI Education Statement:</ins> Injectable medicines for HIV, also called Long-Acting Injectables or LAI, are a new treatment option for HIV that is an alternative to daily pills for some patients. If your HIV care provider determines that you qualify, you can come to the HIV clinic to receive two injections---one injection in each gluteus muscle (each side of the butt)---every 1 to 2 months.

Has your HIV care provider mentioned Long-Acting Injectable medicines to you at any of your previous visits?

Yes

No

I don't know

Do you know if you are eligible to receive Long-Acting Injectable medicines for HIV?

I know that I am eligible

I know that I am <ins>not</ins> eligible

I do not know if I am eligible

If you are eligible or were to become eligible, how interested or disinterested would you be

in receiving your HIV medicine by injection instead of pills?

Very disinterested

Slightly disinterested

Neither interested nor disinterested

Slightly interested

Very interested

Listed below are several possible benefits of using an injectable medicine instead of pills. Please rate each of the following potential benefits of injectable medicines by how appealing or unappealing it is to you.

	Not at all appealing	Slightly appealing	Moderately appealing	Extremely appealing
More privacy: no one will find my pills or pill bottle	○	○	○	○
Don't have to swallow pills	○	○	○	○
Fewer interactions with other medicines	○	○	○	○
Fewer stomach side effects (nausea, vomiting, diarrhea) when not taking pills	○	○	○	○
Don't have to remember to take my medicine every day	○	○	○	○
Don't have to bring pills when traveling away from home	○	○	○	○
More opportunities to see my HIV clinic staff	○	○	○	○

	Not at all appealing	Slightly appealing	Moderately appealing	Extremely appealing
Other (please specify) ___	○	○	○	○

Listed below are several possible drawbacks of using an injectable medicine instead of pills. Please rate each of the following potential drawbacks of injectable medicines by how worrisome, if at all, it is to you.

	Not at all worrisome	Slightly worrisome	Moderately worrisome	Extremely worrisome
Use of a needle to inject the medicine	○	○	○	○
Site of injection in gluteus muscle (butt)	○	○	○	○
Out-of-pocket cost of the medicine	○	○	○	○
Potential side effects of a new medicine	○	○	○	○
Soreness or redness where you receive the injections	○	○	○	○
More frequent trips to the clinic	○	○	○	○
Insurance coverage of the medicine	○	○	○	○
Other (please specify) ___	○	○	○	○

How comfortable, if at all, do you feel initiating a conversation with your HIV care provider about Long-Acting Injectable medicines for HIV?

Very uncomfortable

Somewhat uncomfortable

Neither comfortable nor uncomfortable

Somewhat comfortable

Very comfortable

When asked about changing your HIV medicine, you answered that you are ${e://Field/likely_change} to change your HIV medicine. Now that you have thought about injectable medicines, how likely, if at all, are you to try to make a change to injectable medicine at your next visit?

Very unlikely

Somewhat unlikely

Neither likely nor unlikely

Somewhat likely

Very likely

Note on Eligibility Criteria: Under current FDA guidelines, Long-Acting Injectable medicines are an option only for patents who have an undetectable HIV viral load on oral medicines. Additionally, your HIV care provider must be sure that the virus in your body is not resistant to any of the injectable medicines. Your HIV care provider should be able to check for this.

Demographics

To finish, we have a few questions about you as a person.

What is your age in years?

What is your gender?

Male

Female

Transgender male

Transgender female

Gender fluid

Non-binary

Prefer not to answer

Other

Are you of Hispanic, Latino, or Spanish origin, such as Mexican, Puerto Rican, or Cuban?

Yes

No

Prefer not to answer

What is your race or origin?

White

Black or African American

Asian or Asian American

American Indian or Alaska Native

Native Hawaiian or other Pacific Islander

[ ] Some other race or origin (please specify)

Prefer not to answer

What is your relationship status?

Single

Partnered

Prefer not to answer

What best describes your sexual activity in the last 6 months?

Not sexually active

I am sexually active, and I have had no new partners in the last 6 months

I am sexually active, and I have had one or more new partners in the last 6 months

Prefer not to answer

What is the highest level of education you have completed?

Less than high school

High school degree or equivalent

Some college, but not degree

Completed college degree (includes Associate's, Bachelor's, or any graduate degree)

Powered by Qualtrics
