# Supplementary material for: Engagement in Care, Awareness, and Interest in Long-Acting Injectable Anti-Retroviral Therapy
Source: AIDS Behav. 2024 Jul 1;28(10):3315–25. doi: 10.1007/s10461-024-04423-x (PMC11427500; doi:10.1007/s10461-024-04423-x)
Supplement: Supplementary file 2 — Supplementary Material 2 [file 10461_2024_4423_MOESM2_ESM.docx]

**Engagement in Care, Awareness, and Interest in Long-Acting Injectable Anti-Retroviral Therapy**

Jacob A. Stout BS^1^, Maxwell Allamong PhD^2^, Frances Hung MS^3^, Katherine Link BSN^4^, Cliburn Chan PhD^3^, Charles Muiruri PhD^5,6^, John Sauceda PhD^7^, Mehri S McKellar MD^4^

**Affiliations:**

1. Duke University School of Medicine, Durham, NC, USA
2. Duke Initiative on Survey Methodology, Duke University, Durham, NC, USA
3. Department of Biostatistics and Bioinformatics, Duke University School of Medicine, Durham, NC, USA
4. Division of Infectious Diseases, Department of Medicine, Duke University School of Medicine, Durham, NC, USA
5. Department of Population Health Sciences, Duke University, Durham, NC, USA
6. Global Health Institute, Duke University, Durham, NC, USA
7. Center for AIDS Prevention Studies, Division of Prevention Science, Department of Medicine, University of California San Francisco, San Francisco, California, USA

**Running** **Title**: Interest in Long-Acting Injectable ART

**Corresponding** **Author**: Mehri S McKellar, MD, Duke University, P.O. Box 102359, Durham, NC 27710, (919) 613-6129, [mehri.mckellar@duke.edu](mailto:mehri.mckellar@duke.edu)

Supplemental Figures:

**Supplemental Fig. 1** Reasons Excluding Participants from Participation

**
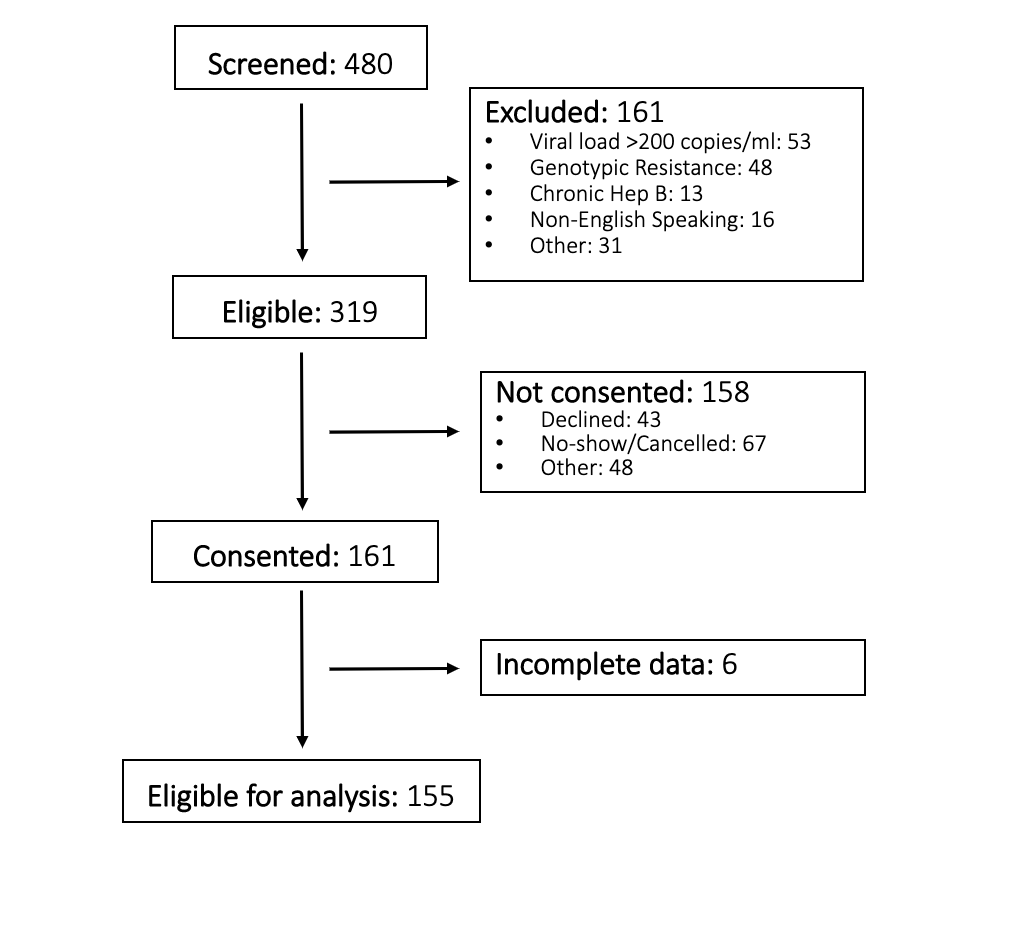
**

Legend: This flowchart summarizes the reasons for participants being excluded from the study. For the excluded individuals, the “Other” category predominantly consists of patients whose provider determined them to be a poor candidate for participation. Of those not consented, the “Other” category consists of patients that research staff were logistically unable to speak with prior to their departure from clinic.

**Supplemental Fig. 2** Reasons Excluding Participants from Regression Analysis

Legend: This flow chart summarizes how the final sample was grouped for regression analyses. The left side of the figure describes our sample for regression analysis of interest in LAI, while the right side of the figure describes the sample for regression analysis of awareness of LAI.
